# Buffalo Pathological Society

**Published:** 1891-01

**Authors:** 


### Society Proceedings.

### BUFFALO PATHOLOGICAL SOCIETY.

STATED meeting October 17, 1890.

Dr. ROSWELL PARK read a paper entitled

### TETANUS AND TETANY.

(Abstract.)

Though these two conditions have such similar names, there exists a vast difference between them as regards their etiological features, and many of their symptoms.

*Tetany* is a neurosis manifesting its effects by tonic spasms, particularly of the extremities and increased mechanical excitability of the peripheral nerves.

It is a disease which is found in those patients who have submitted to total extirpation of the thyroid, and in pregnant and nursing women. It is also sometimes observed in children after exposure to cold, and during some intestinal lesions, as those of typhoid, and with the irritation caused by intestinal parasites. It is, as a rule, endemic, but it may be so diffused in certain parts of the world as to come under the head of an epidemic disease.

Tetany follows as a very serious complication after thyroidectomy (total). Tetanella, idiopathic muscular spasm, carpo-pedal cramps are synonyms which should be understood as such, so that one is not misled into believing that they are distinct diseases or conditions. The patients do not lose consciousness ; and the spasms are those of a functional neurosis, coming on a tolerably well defined rythm or order.

Some observers have claimed that it is due to meningeal hyperemia, and others that it is caused by a primary lesion of the primary cells of the cerebro-spinal tract. A few cases are recorded where the trouble seemed to have developed into chronicity, but, as a rule, all those affected die ; we may say that it is maligant, if we keep the mortality rate in mind.

The first symptoms may come on at once, for example, just as the patient is recovering from the anesthesia, or the disease may be delayed ten days or so, after the exhibition of the cause. Prodromal symptoms often put in an appearance, such as malaise, muscular weakness or stiffness ; the onset, however, may be sudden and come with extreme violence.

There are two characteristic diagnostic signs which can be elicited in this disease, *i. e.* :

Chvostek's ; a tap at the point of exit of the facial nerve sets the muscles of the same side of the face into a spasm ; this sign may be induced without much harm to the patient, which, however, is not true with the second : Trousseau's ; pressure for a few seconds or minutes on the main vessel or nerve supplying a limb causing it to go into general tonic spasms.

The facial muscles are first to be affected, those of the arms following next, while the spasms in the lower extremities may be less severe or be absent entirely. The hands and fingers assume a position similar to that taken in irritation of the ulnar nerve. The fingers are strongly flexed at the metatazso-phalangeal articulation, stiff and straight beyond ; the hand is strongly bent to the ulnar side, and the thumb is closed upon the palm. The muscles of the forearm are firm, and there may be a slight tremor. In the legs we find similar muscular conditions ; they are extended, and the feet and toes are found in plantar flexion. There may be pain in the parts; the temperature is somewhat elevated. Each attack, or seizure, lasts from two to fifteen minutes, and is not of so frequent occurrence as those of tetanus.

Naturally, from so great spasm, we may have dyspnea and cyanosis. In the gravest cases consciousness may be lost; though, as said before, this is not usually the case. Death never occurs in the height of the disease, but usually some days later.

Overwork or great excitement sometimes precipitates an attack. Both sexes suffer from the malady, but females seem the more prone.

To meet indications, it has been suggested to use chloral and morphia, and these remedies have actually been of benefit in some cases. The present writer suggests, inasmuch as several cases have been recorded as having been much improved after profuse diaphoresis, that pilocarpine be exhibited to its full physiological effect.

In relation to its etiological features, much matter has been accumulated, particularly during recent years. The disease supervenes only after total extirpation of the thyroid, not after partial removal.

A great many observers claim that tetany is essentially due to sepsis, or is of a septic nature at least. There are many facts which weigh against the assumption that there is any septic element about the disease, etiologically speaking. It occurs only after total thyroidectomy; is almost unknown after other operations. Experiments on animals have fully borne out what has just been said concerning tetany. Cats and dogs are very prone, and almost without exception fall certain victims to its influences. Sheep, rabbits, and rats are singularly exempt from its effects. If the gland be removed by halves, the usual symptoms ensue only after the second half is excised. During the interval between the two operations the remaining half hypertrophies. If the thyroid be four-fifths removed, tetany follows. It has been claimed that if one-half of the thyroid, or even a smaller portion, be removed and inserted, with proper and due precautions, into the peritoneal cavity, the implanted section will contract adhesions and grow, and that in such a case, if the remainder of the thyroid be then removed, the usual symptoms of tetany do not appear. This has been explained on the ground of the thyroid's function being taken up by the transplanted portion, a theory which is as yet not proven.

Clinically there occurs a disease which is practically the chronic form of tetany, to-wit, myxedema. The connection between myxedema and thyroid disease or removal, has long been hinted at, though until the recent work on tetany was reported, there was not much experimental ground to base this opinion on. The possible existence of accessory thyroids must not be lost sight of, for otherwise certain experiments may seem to vitiate the connection between tetany and loss of the thyroid, whereas they really do not, because of an additional thyroid which may be present and which assumes the duties of the lost organ. Animals, whose thyroids have been wholly removed, show signs of tetany similar to that in man; they are dull, apathetic, have spasms, dyspnea, etc., and die within a few days.

In apes the disease appears very slowly, if the animals be kept warm and protected from loss of bodily heat.

It is supposed that the thyroid has to do with maintaining the amount of *mucin* in the blood at a proper level. Mucin extracted from normal thyroids is highly poisonous when injected into the circulation; after thyroidectomy there appears to be an excess of mucin in the blood and tissues; in myxedema mucin appears in abnormal quantities in the salivary bladder excretion, intestinal contents, blood, and tissues; myxedema patients often suffer from a profuse bronchial catarrh, in which the secretions contain an unusual amount of mucin, and where this catarrh is checked the general condition of the patient depreciates; from all of these facts it is believed that the thyroid has to do with converting the excess of highly deleterious mucin into some non-poisonous material.

In view of these facts, it is assumed that in goitre, when the thyroid is found in a state of so-called myxoid degeneration, that the proper function of this gland has been lost, and that the organ then acts as a store-house for the excess of mucin.

*Tetanus* follows particularly after wounds, but it may occur with an unbroken skin. Some wounds are more liable to be followed by tetanus than others, for example, those of a lacerated or contused nature, and gun-shot wounds. The disease may appear early or late after the infliction of an injury.

It is said that cases have been observed as late as one year after the injury, and it may also appear as early as a few days subsequent to the wound. A wound does not need to be open to permit of the tetanus symptoms pronouncing themselves; it is well known that cases occur after thorough cicatrization of the wound.

It is a malady attacking young or old indifferently; males seem more liable to its ravages, probably from greater chances for injury because of greater exposures. Statistics show that the colored races are sufferers to a larger extent than the whites ; this is particularly true of the West Indies negroes, who have a high rate of mortality from tetanus, and here the children, newly born, are very commonly attacked (the so-called *trismus nascentium*). It is supposed that the greater filth in which these races live, predisposes to the disease. Climate also seems to act as a predisposing factor, those people of warmer climates showing a larger mortality.

It is a disease characterized by tonic spasms of almost universal distribution as regards the muscles involved, those of the face usually showing the first signs. There is a form, the *tetanus hydrophobicus* of the older writers, where the spasms seem unusually severe in those parts supplied by the facial and other cranial nerves. It occurs in those cases where there is much facial injury. When the disease is well established, there seems to be a more or less constant succession of spasms ; a patient may have such an attack, become quiet, and a most trifling irritation, noise, or effort will cause another succession of spasms; even the mere sight of a bright light suffices to accomplish this. In the so-called *tetanus hydrophobicus*, an effort to take a drink will cause a spell of facial contractions to ensue, or tapping on the face may be enough to precipitate the spasm.

Formerly two theories were held to account for this disease ; the nervous, whose advocates claimed that it arose through irritation of the nerves involved in the wounded areas ; and second, the so-called humoral, where the disease was ascribed to a humor or poison in the blood. It is now positively known that the trouble is of germ origin ; a bacillus having been isolated, which fulfills all three postulates necessary to prove a germ's connection with a disease as a causative agent. The bacillus is an *obligate anærobe* ; that is to say, a germ which only grows when all oxygen is excluded, and this is one factor which has prevented its recognition earlier.

There are many peculiarities connected with the bacillus of tetanus. For example, it is never found in any of the tissues of a patient, except in the immediate neighborhood of the wound or injury. It seems to excite a most powerful poison —*ptomaine*— which apparently has the power of propagating itself along the cerebro-spinal nervous tract. Cauterization or excision of the wound — the focus of infection — does not terminate nor even delay the symptoms. There have been a number of *ptomaines* isolated from the tissues of patients or animals suffering from tetanus, and from cultures of the bacillus, but none of them come up to the extreme virulence of the disease, and the probabilities are that there will yet be another ptomaine isolated, which will be even more deadly than any heretofore discovered.

Dr. Park exhibited a guinea-pig that he had inoculated with a pure culture of the bacillus of tetanus sixty hours previously, the animal displaying characteristic spasms of the muscles of the posterior part of the body. (The guinea-pig lived twelve hours longer, and died displaying universal tetanus.) A cat was also exhibited whose thyroid gland had been completely extirpated five days before; this animal displaying the characteristic tremor and other signs, alluded to by the lecturer, as completely as the length of time elapsing since operation permitted. It also died after a few days of acute tetany.

